# An Endoscopic Approach to a Pouch Stricture After Failed Needle Knife Stricturotomy

**DOI:** 10.14309/crj.0000000000002161

**Published:** 2026-06-02

**Authors:** Ridhima Kaul, Arjun Chatterjee, Renan Prado, Hassan Siddiki

**Affiliations:** 1 Department of Internal Medicine, Cleveland Clinic Foundation, Cleveland, OH; 2 Department of Gastroenterology, Hepatology and Nutrition, Cleveland Clinic Foundation, Cleveland, OH

## CASE REPORT

Anastomotic strictures may occur after ileal pouch–anal anastomosis, although strictures between the pouch body and proximal limb are uncommon. Balloon dilation with or without needle-knife stricturotomy is first-line therapy, but dense fibrosis may lead to early restenosis.^1–3^ We report the use of bipolar radiofrequency (RF) scissors for a refractory pouch anastomotic stricture.

**Figure 1.**
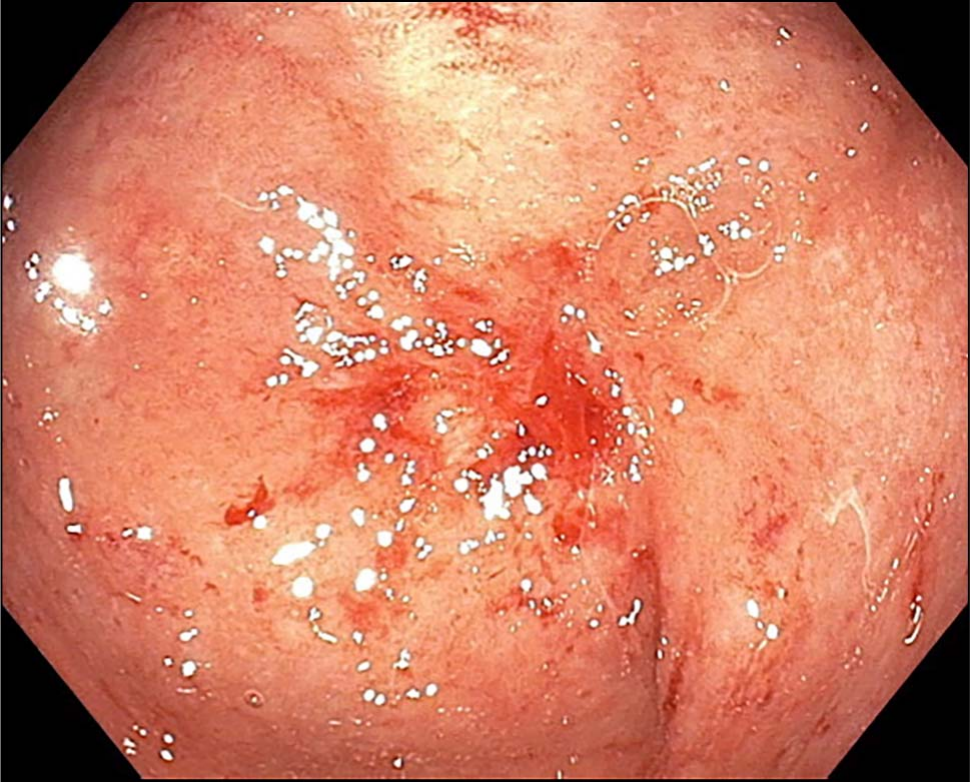
Flexible pouchoscopy demonstrated that the pouch was obstructed and a central scar formation.

**Figure 2.**
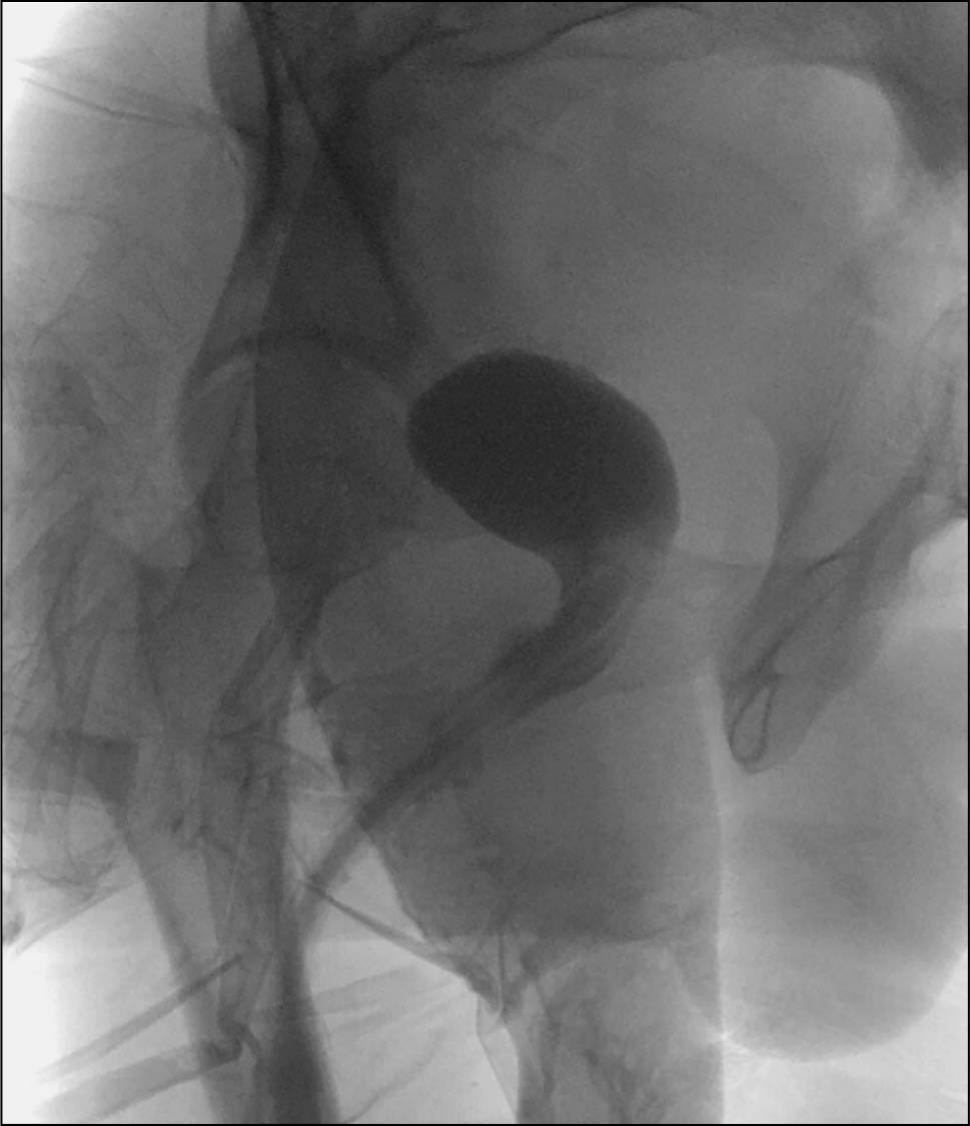
Follow-up pouchogram and gastrograffin study demonstrated severe restricturing with failure of contrast to enter the proximal pouch.

**Figure 3.**
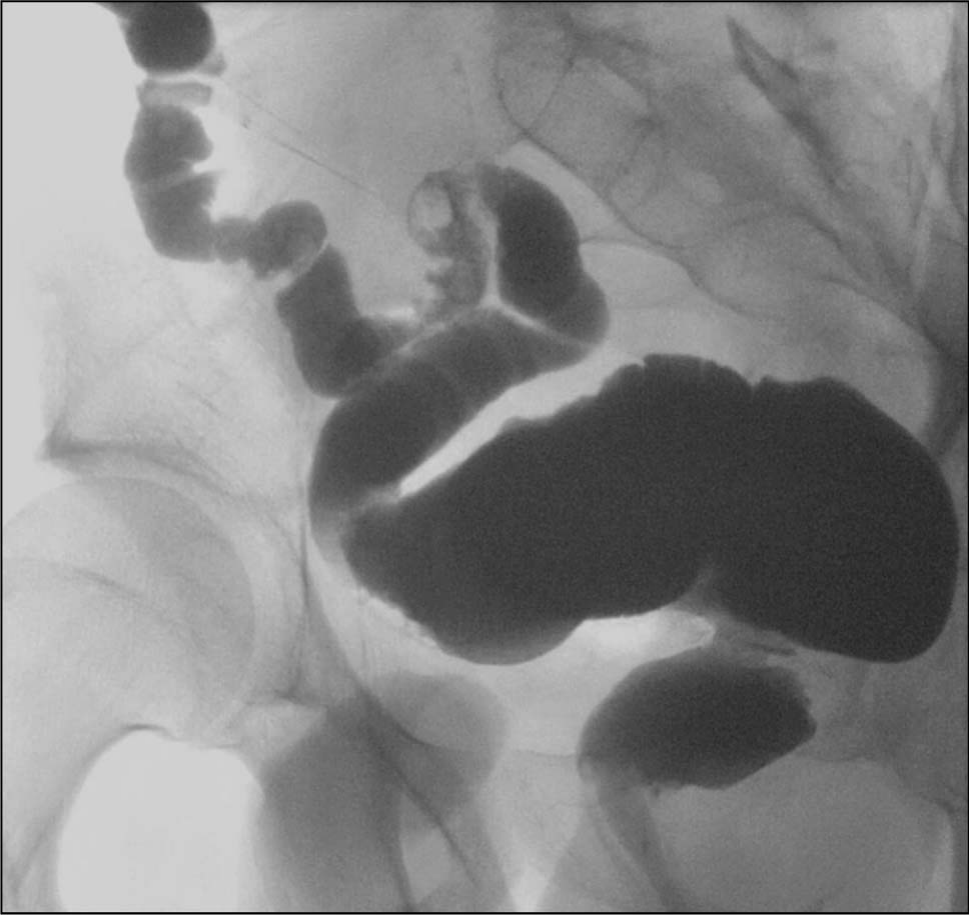
Follow up pouchogram showed mild narrowing of the ileoanal anastomosis and the pouch distending normally with contrast flowing through the pouch and to the stoma without obstruction and without leak.

A 22-year-old man with ulcerative colitis underwent 3-stage ileal pouch–anal anastomosis and developed a fibrotic stricture between the pouch body and proximal limb, preventing ileostomy reversal (Figure 1). Balloon dilation and needle-knife stricturotomy achieved transient patency, but contrast enema demonstrated rapid restenosis (Figure 2). Bipolar RF scissor stricturotomy was performed using 3 radial incisions (3–4 mm depth) in cut mode (400 kHz, 15–35 W), followed by balloon dilation to 15 mm. Mild bleeding was controlled endoscopically. Follow-up imaging confirmed restoration of luminal continuity without leak (Figure 3). The patient underwent successful ileostomy reversal and remained asymptomatic at 6 months (Video 1).

Bipolar RF scissors provide a precise salvage option for fibrotic strictures refractory to standard therapy, enabling controlled energy delivery and durable luminal patency.^4,5^

## DISCLOSURES

Author contributions: R. Kaul, A. Chatterjee, R. Prado: drafting the article. H. Siddiki: final approval of manuscript. H. Siddiki is the article guarantor.

Financial disclosure: None to report.

Informed consent was obtained for this case report.
